# Expanding capacity in mental health research in intellectual disabilities

**DOI:** 10.1192/bjb.2020.117

**Published:** 2021-12

**Authors:** Angela Hassiotis, Peter Langdon, Ken Courtenay, Ian Hall, Bryn Lloyd-Evans, Renee Romeo, Athanasia Kouroupa, Vicky Crossey, Laurence Taggart

**Affiliations:** 1 University College London (UCL), UK; 2 University of Warwick, UK; 3 Chase Farm Hospital, London, UK; 4 East London NHS Foundation Trust, UK; 5 University College London (UCL), UK; 6 King's College London, UK; 7 University College London (UCL), UK; 8 NHS Lothian, UK; 9 Ulster University, UK

**Keywords:** Consent and capacity, intellectual disability, service users, psychosocial interventions, randomised controlled trial

## Abstract

Although the research base on mental health in intellectual disabilities is advancing, there are long-standing barriers that hinder successful completion of funded studies. A variety of stakeholders hold the key to mitigating the challenges and arriving at sustainable solutions that involve researchers, experts by experience, clinicians and many others in the research pathway. Lessons learned during the COVID-19 pandemic can also contribute to improvements in the conduct of research in the medium to long term. People with an intellectual disability and mental health conditions deserve high standards of evidence-based care.

Research evidence is essential in supporting professional decision-making for the benefit of patients across health and social care. The benefits of participation in research include better outcomes and more efficient use of resources, with harmful or unhelpful treatments being phased out.

A major driver for funded applied health and social care research is the increase in research capacity and the completion of high-quality studies on priority topics that have been identified by stakeholders, including scientists and the public. In the UK, the substantial annual investment of more than £1 billion by the National Institute for Health Research (NIHR) supports both projects spanning the range of methodologies and the infrastructure to underpin the endeavour.

Recognising the challenges of investing and supporting research in health and social care organisations, NHS England and the NIHR published a joint report^1^ that included 12 actions that would help to relieve the bottleneck many chief investigators encounter at the setting-up and recruitment phases. Two major stumbling blocks at the time were setting excess treatment costs with regard to treatment delivery, and research governance; the latter ranges from ethical approval to assessment of local capacity and capability in agreeing recruitment targets.

Although progress has been made, these issues, which are common in research activity across many different health and social care domains, have not completely resolved 3 years on. In this editorial we address a variety of both barriers to research and facilitators of research, with a specific focus on research in intellectual disability services. We argue that such problems may be relevant in research in other hard-to-reach populations who may or may not have cognitive impairment.

Since the launch of the NIHR in the UK in 2006, there has been an increase in the number of funded studies investigating a variety of research questions in intellectual disabilities, including developing, adapting and testing interventions in randomised controlled trials (RCTs). The majority of these are trials of psychosocial interventions. However, the portfolio of studies remains small compared with other fields of medicine, estimated at 1.4% of all NIHR-funded studies.^2^

People with intellectual disabilities (global developmental delay evident in childhood that affects adaptive functioning) account for approximately 2% of the population in the UK and are more likely to suffer health-related multimorbidity, higher and earlier mortality and face significant inequalities.^3^ Many lack capacity and therefore decisions about their participation in research depend on families’ and paid carers’ understanding and attitudes towards research projects and research processes. This is because they may be called on to act as consultees to enable participation of those most vulnerable. It is therefore essential that people lacking capacity should also be able to take part in and benefit from research specific to people with intellectual disabilities, with appropriate safeguards as mandated in the Mental Capacity Act 2005 or equivalent permissions internationally.

## Barriers

Despite the amount of research conducted over time, many challenges that have been reported previously still remain and have an impact on the completion of studies. Lennox et al^4^ described identification of substantial numbers of participants, frequent need for substituted decision-making, occasional limited literacy of both person and carer, and organisational gate-keeping practices as significant barriers to recruitment in an RCT of health checks in Australia. More recently, a systematic review of 53 papers reporting RCTs in people with intellectual disabilities published between 2000 and 2017^5^ identified similar barriers in recruiting to target, participant treatment preferences, engaging with stakeholders, obtaining consent and staff turn-over. Optimistically the authors concluded, ‘conducting RCTs with people with cognitive disabilities can be challenging, however, with reasonable adjustments, many of these barriers can be overcome’.

People with intellectual disabilities are excluded from research that may be relevant to their health vulnerabilities,^2^ as well as being sceptical about the impact of it on their lives. This is illustrated by the Research Voices project,^6^ which revealed a number of serious concerns that parents of people with profound and multiple disabilities harbour about research, such as mistrust of health professionals seeking participants for studies, the emotional and time burden of research assessments, frustration with not knowing the findings or findings not translating to real improvements in practice.

Research infrastructure brings its own multifaceted challenges. The role of clinical research practitioners (CRPs) (who are National Health Service (NHS) based and able to recruit from services directly) is not fully understood by intellectual disability services and there is significant variation among the operations of clinical research networks across the different UK countries. Therefore, professionals in the services may be asked to undertake recruitment in addition to an already busy clinical role. Further, data guarantors are frequently local authorities, who are providers of social care services not directly connected to the NHS in England. Finally, intellectual disability services that are located outside NHS structures have fewer opportunities to be informed of ever-evolving research processes, thus remaining unable to utilise available resources to assist them in incorporating and supporting research in their day-to-day practice.

Clinician factors are also important in maintaining non-engagement in research activities, including older age, being male and working in the private sector.^7^ Oliver-Africano et al^8^ identified beliefs about drug efficacy, potential ethical conflicts in medication trials and multidisciplinary team processes as having adversely affected recruitment to a clinical trial of antipsychotics in adults with intellectual disabilities. Delays associated with any of these factors inevitably matter in completing studies that depend on time-sensitive research contracts and are likely to hamper the validity of the research findings if there is underrecruiting.

Finally, clinicians and scientists in general may not make sufficient effort to include participants from underrepresented groups in their studies, thus perpetuating the limited access of people with intellectual disabilities in research. This is particularly important for diseases where it has been demonstrated that there is excess morbidity and/or mortality in this population.^9^

## Facilitators

Prioritisation of research is likely to confer benefits to both health and care organisations, as shown by views reported in a review of engagement in research:^10^

‘The wider review demonstrated […] how collaborative and action research can encourage some progress along the pathway from research engagement towards improved health-care performance. There is also evidence that organisations in which the research function is fully integrated into the organisational structure, out-perform other organisations that pay less formal heed to research and its outputs.’

These lessons, although not specific to intellectual disabilities, are relevant in this context as presenting a justification for embracing research by the multitude of service configurations delivering care to this population.

In other changes to research governance, the new Health Research Authority has halved the time needed to obtain regulatory approvals,^11^ although other milestones along the research pathway remain areas of concern.

Although the funding for research in intellectual disabilities may be lower than what is essential for investigating the increased morbidity, mortality and the health inequalities seen in this population group, there is an emerging cohort of completed high-quality studies. There are also many committed researchers, healthcare service professionals and other staff whose enthusiasm and problem-solving capacity signal their willingness to engage with the process. These may further promote interest in research by influencing national clinical practice and through targeted dissemination, including to people with intellectual disabilities and their carers. Clinician familiarity with academic work, peer support and support from management are also likely to increase positive attitudes towards research. Research that is seen as arising out of patient concerns and that could lead to tangible benefits in interventions and care improvements is also likely to be supported.^6^

Recently the NIHR Dissemination Centre published a themed review on intellectual disability research.^12^ More than showcasing the funded projects, it highlighted the meaning of the findings for the care that people with intellectual disabilities and their family carers receive. In addition, the collection of studies included in the review demonstrate that funded research can be conducted successfully in the field of intellectual disabilities but that all the studies have had significant involvement of people with intellectual disabilities and their family carers throughout. These studies are examples of good practice that can be shared between researchers, people with intellectual disabilities, their carers and charities supporting them.

## Solutions

A primary area for mitigation lies in health and social care professionals’ and people with intellectual disabilities’ conviction of the importance of research and its wider contribution to lives and well-being. A recent course^13^ devised to train people with intellectual disabilities in research methods suggests that learning about conducting research and driving the research process is feasible. Such courses could increase the number of suitably trained people with intellectual disabilities who could be recruited to work as researchers in various projects.

Incentives for encouraging donation of time to research activities by family and paid carers may improve uptake and retention, alongside other strategies. Increasingly, experts by experience are being asked to interpret and comment on research findings and this is a way of increasing familiarity with research processes, as well as consumer feedback.

The well-intended efforts of paid carers to protect vulnerable adults if they lack capacity, including the personal data protection regulations, often stifle participant recruitment. In England and Wales, this could be addressed by a revision of the Mental Capacity Act 2005. Heywood et al^14^ outlined how the Act is predominantly focuses on treatment and decision-making within a ‘best interests’ framework, rather than on research where decisions are not made using that framework. The sections of the Act governing research do not effectively balance protection and empowerment, and researchers may be reluctant to include participants who lack capacity in research projects.

The research community may also need to take some responsibility in providing solutions to the present challenges. Being clear about what the findings mean to the wider group of people with intellectual disabilities, proactive dissemination strategies and other activities in engaging the public with research are paramount in moving forward. Research aims to shape service delivery and to translate advances in science into measurable benefits for the population at large. It is questionable whether consumers consider research findings relevant to their health and whether those who commission services apply research findings to enhance clinical effectiveness and value for money.

An example of fostering closer links between clinicians, academics and people with lived experience of intellectual disability is the newly formed RADiANT consortium, a platform that works to increase health and social care staff's awareness of research and develop research skills and capacity. The consortium is focused on mental health and behavioural problems in intellectual disabilities, autism and other neurodevelopmental conditions (see radiant.nhs.uk for more information). So far it has produced guidance on how to manage the COVID-19 pandemic in different mental health settings and has delivered several educational activities. Its wider impact remains to be established.

Professional bodies across all professions must also promote research-related objectives in training curricula and on public-facing forums such as websites and newsletters.

Strengthening health and social care links is an area for further development, especially as social care is identified by NIHR as a domain for research investment. This means extension of the research infrastructure to reach the neglected care sector, which is central to accessing individuals to take part in research activities as proxy informants.

The coronavirus pandemic has shown that, while continuing to endorse ethically conducted research, it is possible to do so at pace.^15^ It will be important to remember those lessons as we are coming out of the pandemic and in the event of future public health emergencies. In particular, they can inform how to carry out remote research assessments and interviews with participants with intellectual disabilities and ensure that the voice of experts by experience remains central to research activity. During the pandemic, people with intellectual disabilities have been disproportionately affected in both their health and social care needs and require high standards of support in both. We must be able to reassure them and their families that being partners in research pays off in achieving those standards.

## About the authors

**Angela Hassiotis**, FRCPsych, PhD, is Professor of Psychiatry of Intellectual Disabilities in the Division of Psychiatry at University College London (UCL), and a consultant psychiatrist with Camden and Islington NHS Foundation Trust, London, UK. **Peter Langdon**, PhD, is a professor in the Centre for Educational Development, Appraisal and Research at the University of Warwick, and an honorary consultant clinical and forensic psychologist and approved clinician with Coventry and Warwickshire Partnership NHS Trust, UK. **Ken Courtenay**, FRCPsych, is a consultant psychiatrist at Chase Farm Hospital, London. **Ian Hall**, FRCPsych, MPhil, is a consultant psychiatrist with East London NHS Foundation Trust. **Bryn Lloyd-Evans**, PhD, is an assistant professor in the Division of Psychiatry at UCL. **Renee Romeo**, PhD, is a senior lecturer in health economics at the Institute of Psychiatry, Psychology and Neuroscience, King's College London. **Athanasia Kouroupa**, MSc, is Study Manager in the Division of Psychiatry at UCL. **Vicky Crossey**, MRCPsych, is a consultant psychiatrist with NHS Lothian, Edinburgh, UK. **Laurence Taggart**, PhD, is an assistant professor in the School of Nursing at Ulster University, UK.
